# Establishment and reinforcement of the national reference centers for human microbiology in Belgium

**DOI:** 10.1186/0778-7367-70-16

**Published:** 2012-06-22

**Authors:** Gaëtan Muyldermans, Amber Litzroth, Geneviève Ducoffre, Sophie Quoilin, the Medical-Technical Advisory Board

**Affiliations:** 1 Institute of Public Health, Public Health and Surveillance, J. Wytsmanstreet 14, 1050, Brussels, Belgium

**Keywords:** National reference centers, Public health microbiology

## Abstract

**Background:**

Microbiology reference laboratories are critical in the development of high-quality clinical and public health services. In Belgium, the reference laboratories performed their activities on a voluntary basis and lacked a legal status.

**Methods:**

Pathogens or groups of pathogens necessitating a national reference center (NRC) were prioritized based on diagnostic and epidemiologic relevance. Terms of reference for each of these pathogens were developed.

**Results:**

Recently, 40 NRCs for different pathogens or groups of pathogens have been installed in Belgium to fulfill the following core functions: offering reference diagnostics, collecting reference materials, sharing information and scientific advice, participating in national and international networks, collaborating with research workgroups, and contributing to surveillance activities.

**Conclusions:**

These NRCs are important focal points of the national and international network in public health microbiology.

## Background

In Belgium most clinical microbiological analyses are reimbursed by the health care insurance and is coordinated by the Belgian National Institute for Health and Disability Insurance (RIZIV/INAMI). A coded list, commonly called nomenclature [1], of medical services including these clinical microbiological analyses is available. However, some activities performed by reference laboratories are not included in this coded list and requires an additional reimbursement system.

In the past, multiple laboratories in Belgium fulfilled these reference activities for several pathogens and thus supporting patient care by the diagnosis of rare diseases or by the confirmation of a diagnosis. These laboratories also contributed to the public health by the detection of new threats and outbreaks, the identification of the source contaminant and the monitoring of strain characteristics.

Historically, these laboratories initiated their reference activities based on scientific interest. They developed into reference laboratories on a purely voluntary basis and were informally recognized as such by the microbiologists, clinical and public health physicians in Belgium. However this was without a legal status and financial compensations.

Given the lack of financial incentives for the accomplished reference laboratory activities, the risk for reducing or even stopping the reference activities became a major issue for both patient care and public health.

Some efforts have been made to compensate some laboratories for their reference activities, as for the 7 ‘AIDS reference laboratories’, a national program for a single pathogen (HIV), for the 18 ‘Centers for Molecular Diagnostics’ for their contribution to the molecular diagnostic assays, and for the ‘Reference Laboratory for the Diagnosis and Treatment of Infections and Tropical Diseases’. However, no overall project was established for all the other pathogens for which reference activities were still performed by multiple laboratories.

The emergence of new strains, pathogens, resistance profiles and the development of new treatments necessitate a constant monitoring of their evolution [2]. Collecting the individual data to monitor this evolution contributes to an improved public health and allows also fulfilling the international obligations.

Moreover, the World Health Organization (WHO) and the European Centre for Disease Prevention and Control (ECDC) requested an increasing number of epidemiological data for multiple pathogens (such as Polio virus, Rabies virus, *Clostridium botulinum*, …) which should be collected by a recognized reference center [3,4].

Neighboring countries such as France [5], Germany [6,7], and the United Kingdom installed national reference centers and they experienced the benefit for patient care as well as for public health.

For all the reasons mentioned above, the federal Scientific Institute of Public Health (WIV-ISP) took the initiative to reinforce the Belgian reference laboratories through a step-by-step process and to create a legal framework ensuring the functioning of the network of national reference centers (NRC).

Here we describe the prioritization and selection of the pathogens, the coordinated approach of the selection of reference centers and their current and future tasks at a national and international level.

## Methods

### Partners/stakeholders of the project

The royal decree for establishing the reference centers for human microbiology became available on 9 February 2011 (published 1 march 2011). The royal decree described the legal and financial framework, the duration of the conventions, the different partners and stakeholders involved and the general terms of reference including the expected tasks of the NRC.

**Figure 1:**
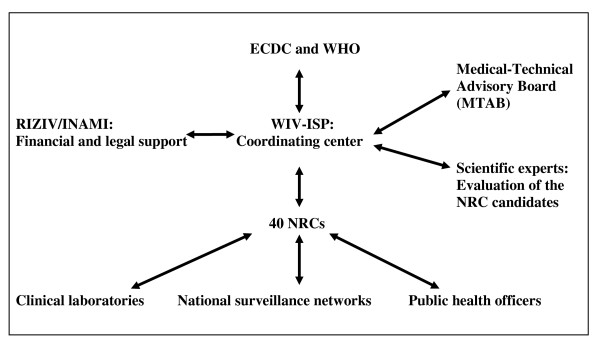
**Structural organization of the partners of the National Reference Centers (NRC) project demonstrating their relationship.** The Scientific Institute of Public Health (WIV-ISP) receives scientific advice and financial and legal support from respectively the “Medical-Technical Advisory Board (MTAB)” and the “Rijksinstituut voor ziekte- en invaliditeitsverzekering / Institut national d’assurance maladie-invalidité (RIZIV/INAMI)”. The selected NRCs work in close collaboration with other partners to collect appropriate data, samples and reference materials. Data are transferred from the NRC through the WIV-ISP to the ECDC (European Centre for Disease prevention Control) or WHO (World Health Organization).

The partners or stakeholders of the project and their relationships are represented in Figure 1.

The financial and the legal support are provided by the national RIZIV/INAMI.

The Medical-Technical Advisory Board (MTAB) has a scientific and advisory role and is composed of the different stakeholders of the project: representatives of the sponsor (RIZIV/INAMI), members of the university community, of the regional and federal health authorities, of the national laboratory surveillance network, and of the medical trade union.

The MTAB developed the list of pathogens necessitating a NRC and reviews this list annually based on predefined criteria and in function of the epidemiological and diagnostic needs. Similarly, the MTAB provided advice in the development of the terms of reference for each pathogen. The MTAB finally advised on the selection of the NRC candidates based on the evaluation by the scientific experts.

The individual evaluation of the NRC candidates by the scientific experts was based on the application documents and the predefined evaluation criteria. These experts were selected based on their expertise in the field of microbiology, epidemiology or public health for the particular pathogen. A total of 3 experts were foreseen for each pathogen or group of pathogens, among which at least 2 international experts.

The coordinating center of the project is located at the national Scientific Institute of Public Health (WIV-ISP) and has two missions: the administrative coordination of the project (i.e. preparatory work for the MTAB, general follow-up of the conventions, management of the budget) and a scientific support for public health related aspects (i.e. assessment of the epidemiologic quality of the data, implementation of data transfer tools, public health expertise).

## Results

### Selection criteria pathogens

As in our neighboring countries [8-10], infectious diseases were prioritized. In selecting which pathogens necessitated a NRC, 3 domains were defined based on their type of importance in public health. A first domain consisted of the pathogens with an impact on nosocomial transmission and antimicrobial resistance (e.g. extended-spectrum beta-lactamase (ESBL) and carbapenemase-producing *EnterobacteriaceaeClostridium difficileStaphylococcus aureus*). Pathogens from the second domain had an impact on surveillance, warnings and actions in the field of public health (e.g. *Bordetella pertussis*, rotavirus, *Haemophilus influenzae*, poliovirus). This domain includes the pathogens linked to vaccine-preventable diseases, diseases/pathogens requiring direct public health action by the health inspection (e.g. chemoprohylaxis) and pathogens subject to international commitments (e.g. eradication objectives). The last domain includes pathogens for which it is important that the diagnosis or confirmation takes place in one center where all necessary expertise is concentrated (e.g. rabies virus, congenital infections, tick born encephalitis virus, West Nile virus, *Clostridium botulinum*). Included in this domain are the rare pathogens, the ones that are difficult to diagnose and those requiring a high biosafety level (BSL) such as emerging pathogens, or pathogens/diseases requiring a diagnostic confirmation in a specialized center. Some pathogens belonged to more than one domain.

Forty (40) pathogens (Table 1) or groups of pathogens were prioritized and selected by the MTAB based on an importance factor varying from moderate to high according to following criteria: burden of disease, severity, mortality, epidemiological dynamics including outbreak potential and emerging potential, information need for international duties and public health attention, health gain opportunity including preventability and treatability.

**Table 1:** List of selected pathogens

**ID**	**Pathogen(s)**
**1**	Antibiotic resistant *Pseudomonas* and *Acinetobacter*
**2**	*Bordetella pertussis*
**3**	*Borrelia burgdorferi* (Lyme disease)
**4**	*Brucella* spp.
**5**	*Burkholderia cepacia* complex
**6**	*Campylobacter*
**7**	*Clostridium botulinum* and *Clostridium perfringens*
**8**	*Clostridium difficile*
**9**	Congenital infections: *Toxoplasma,* rubella, cytomegalovirus and parvovirus B19
**10**	* Corynebacterium diphteriae*
**11**	*Coxiella burnetii, Rickettsia, Anaplasma (Ehrlichia)*
**12**	*Enterococci*
**13**	Enteroviruses including polioviruses and parechoviruses
**14**	ESBL + Carbapenemase producing *Enterobacteriaceae*
**15**	*Haemophilus influenzae*
**16**	Hantavirus
**17**	*Helicobacter pylori*
**18**	Hepatitis B, C, D and E viruses
**19**	Influenza virus
**20**	*Legionella pneumophila*
**21**	*Listeria monocytogenes*
**22**	Measles virus + rubella
**23**	*Mycobacterium* spp.
**24**	Mycosis
**25**	*Neisseria meningitidis*
**26**	Noroviruses
**27**	Rabies virus
**28**	Respiratory pathogens: adenovirus, coronavirus including SARS, human parainfluenza virus, *Mycoplasma pneumonia*e, *Chlamydia pneumoniae,* respiratory syncytial virus (RSV), human metapneumovirus (HMPV)
**29**	Rotavirus
**30**	*Salmonella/Shigella* spp.
**31**	Shiga-toxins producing *E. coli* (STEC)
**32**	*Staphylococcus aureus*
**33**	STI: *Treponema pallidum, Chlamydia trachomatis, Neisseria gonorrhoeae*
**34**	*Streptococcus agalactiae*
**35**	*Streptococcus pneumoniae* invasive
**36**	*Streptococcus pyogenes* invasive
**37**	Tick-borne encephalitis
**38**	*Vibrio cholerae* and *Vibrio parahaemolyticus*
**39**	West Nile virus (arboviruses)
**40**	*Yersinia enterocolitica* and *Yersinia pseudotuberculosis*

Corresponding addresses of the NRCs are available through the website of the project (http://nrchm.wiv-isp.be).

Within these 40 pathogens, five groups of pathogens (sexually transmitted infections (STI), respiratory pathogens, congenital infections, hepatitis and *Salmonella/Shigella* spp.) were defined, grouping different pathogens causing similar symptoms or having similar infection routes. Five other groups (e.g. antibiotic resistant *Pseudomonas* and *Acetinobacter*, *Coxiella brunetii/Rickettsia/Anaplasma*, ESBL and carbapenemase-producing *Enterobacteriaceae,* measles virus and rubella virus, and mycosis) were defined based on the biological similarities of the concerning pathogens. For these groups it is important that the different activities are concentrated in one center. Therefore candidate reference centers had to postulate for the entire group.

Thirty (30) pathogens were selected for a single species or for multiple species belonging to the same species group.

### Description of the terms of reference

Common tasks and necessary qualifications for all pathogens were listed in the general terms of reference. In addition, specific tasks were determined for each pathogen and listed in the specific terms of reference. These specific tasks took into account the particular health problems linked to the pathogen or the group of pathogens.

These terms of reference formed the framework for the commitments of the laboratories and for any evaluation of their experiences and performances by the evaluating group of experts and the selection by the MTAB. Therefore, the terms of reference were a guide for completing the application forms by the candidate laboratories.

### Application phase

The information concerning the call for tender was communicated to the existing reference laboratories, the clinical biology labs, the deans of the faculties of medicine of the Belgian universities, governmental and non-governmental laboratories.

The necessary information on the candidates was collected through an application form. This application form gathered information concerning relevant topics such as expertise in the diagnosis of the infectious disease, team facilities, participation in national and international networks, quality assurance and management, participation in surveillance networks and outbreak investigations, and services offered to routine labs.

### Evaluation and selection procedure

In order to guarantee an objective selection of the NRC candidates, the application files were evaluated by 3 independent experts, of whom at least one expert in epidemiology or public health and at least two foreign experts. These experts gave individual scores on the individual topics described in the application phase. Experts were allowed to abstain from scoring items for which they felt they lacked the necessary expertise.

The MTAB formulated its advice on the designation of the reference centers, based on these evaluations.

## Discussion

### The Belgian NRC in a Belgian context

First, the activities of the NRCs are of interest for the individual patient by improving the patient therapy choice, the confirmation of a screening diagnosis, or the typing of the germ allowing a specific therapy. Although difficult to measure financially, a precise and quick diagnosis makes it possible to avoid or reduce inappropriate or expensive treatments, and to reduce the period of incapacity. Similarly, the detection of (specific) resistance to antimicrobials makes it possible to use effective antibiotics, antifungals or antivirals in disease treatment or prevention. The NRCs use state-of-the-art validated laboratory methods and are able to deliver accurate confirmation of diagnostic results within described timeframes.

On the other hand, the implementation of the NRC will also have an impact on the public health for numerous reasons.

First, they will alert the medical authorities in time in the event of abnormal phenomena, such as the appearance of emerging or rare diseases, the early detection of outbreaks or epidemics, the abnormal increase in the incidence or the virulence of specific pathogens and identifying the sources of infection and the increased risk.

Second, they will assist the medical authorities by bringing a specific expertise in these pathogenic agents and by providing information allowing the adaptation of preventive (e.g. vaccine calendar) and curative (e.g. diagnosis, use of antibiotics) measures.

Third, they will take part in the monitoring of the infectious diseases at the national level by the follow-up of their evolution and characteristics (resistance to antimicrobials, biotyping, genotyping, virulence), as well as by detecting and analysing nosocomial infections.

Finally, they will take part in the international monitoring alert systems of WHO and ECDC in close cooperation with the WIV-ISP.

Furthermore, the implementation of a centrally coordinated network through the national WIV-ISP has an added value for several reasons. Firstly, it allows a central data collection and ensures the data flow and communication to different authorities and public agencies at different levels (European, national and regional).

The installation of one NRC per pathogen rather than multiple reference laboratories operating at different locations for keeping the balance between linguistic, political or governmental versus academic or private, improves the effectiveness of the NRC activities by the geographic coverage of its service. Since the disease surveillance not only includes the collection of data but also compilation, consolidation and analysis of data and inference, the implementation of national coordinated centers are an added value by bringing all expertise together. This expertise in specific pathogens will be shared with the relevant stakeholders. This can include technical advice on methods and procedures, and scientific support for and advice on the interpretation and the relevance of laboratory findings to relevant public health authorities or routine laboratories.

In case a BSL3 facility is necessary for the diagnosis of the particular pathogen, the centralization of the NRC activities makes the funding of the BSL3 facilities more viable.

Also, with the nomination of one NRC per pathogen, the pubic health authorities gave the laboratory a national spokesman role which reinforces their scientific recognition. They will promote the EU harmonization and standardization of key reference testing methods and the timely reporting of epidemiologic data. They will collaborate and interact with research programs on pathogenicity, drug resistance, epidemiologic determinants of human pathogens, presence of vector-borne pathogens in animals or molecular surveillance programs. They are at the forefront of technological and scientific development in their field of expertise particularly in areas relevant to public health action.

Similarly, the reference center plays a key role in the collection of relevant reference materials. These can include international reference strains and a representative strain collection of the Belgian circulating strains as well as sera and genetic materials to evaluate new assays. This collection of relevant reference material is to be shared with laboratories and organizations that require such materials for the varied purposes of quality assurance systems, method evaluation and validation.

### The Belgian NRC in a European context

As in other EU reference laboratories, the Belgian NRCs are not limited to a subtask for clinical laboratories or a support function to the public health in general but rather perform a front line work in terms of monitoring, alerting, responding to crisis situations and supplying scientific advice.

Defining the roles and responsibilities of the Belgian NRCs in line with those in other EU countries will help to create a more stable and sustainable laboratory function across Europe [4]. The NRCs will therefore participate in EU working groups to obtain agreements on collaborations for cross-section and cross-border activities and to formulate the appropriate responses to rare or emerging diseases. Sharing expertise, additional technical and scientific advice, support and training in methodology with other member states will reinforce the local capability [11]. The NRCs could also help in establishing protocols, model agreements and standard operating procedures (SOP’s) at the EU level. The participation in a network of laboratories, able to help in surge situations, will be stimulated.

Furthermore, a harmonization for typing methods to achieve comparable results will be recommended to the NRCs. These allow the identification of cross-border outbreaks and zoonotic transmission. They will also allow to report accurate and comparable microbiology data to international surveillance systems in compliance with case definitions and surveillance protocols.

By requiring an ISO15189 accreditation from the NRCs, they will be conform to internationally agreed quality standards. This will be particularly useful in case of involvement in cross-border network activities.

### Implementation of a new reporting system

An efficient and effective manner to communicate the aggregated information back to the data providers (NRCs and clinical laboratories) will be put in place by the WIV-ISP.

The WIV-ISP developed a web-based database allowing the NRC to report and consult the individual data from the patients, the samples and the results.

The variables to report to this database are based on the requirements of international organizations [12] (e.g. ECDC, WHO) and national and regional health authorities [13]. In function of the epidemiologic requirements, the timing of reporting can range from real-time to yearly.

These data will be used for automated and regular (weekly, monthly, yearly) reporting and, in case of an epidemic or outbreak, also for automated alerting based on defined threshold levels [14].

### Determination of the selection criteria for samples

Minimum testing requirements for informative laboratory-based surveillance will be further elaborated. Evidence-based technical guidance on appropriate sampling and microbiological testing for diagnosis of infection and further characterization of human pathogens of public health relevance will be provided.

Defining selection criteria for samples by the NRC and verifying whether the sample fulfills the criteria are crucial steps in avoiding redundant analyses. These selection criteria, the request forms, the transportation conditions and the turnaround time for analysis are available for all routine laboratories through the website of the NRC [15].

As the available resources are limited, the volume of activity is the main item that needs to be controlled. For surveillance, the minimum investigation necessary to obtain representative results should be the starting point. Therefore, the NRC needs to estimate the minimal fraction of the received samples necessary to obtain a representative result for the population at risk.

### Preparedness for emerging diseases

Preparedness for emerging diseases is a prerequisite to handle future threats caused by emerging pathogens and epidemic diseases. Therefore, a risk mitigation of a possible emerging disease should be performed by the simulation of incidents (i.e. increasing incidence, new pathogens and new strains) with a significant public health threat. An extra budget was made available for emerging diseases. The NRC should be prepared in case an emerging disease occurs in terms of the infrastructure, strain identification and typing, cross-sector work, specialist capabilities, and the choice of response (international, national, regional).

## Conclusions

Facing new threats due to a changing world, the launch of the NRCs will have an impact on the patient care by performing laboratory diagnosis, pathogen characterisation and susceptibility testing. Similarly, the NRCs will contribute to the microbiology surveillance and outbreak investigations by the availability of different typing techniques.

The implementation of the NRCs allows the development of an integrated surveillance and epidemic intelligence of antimicrobial resistance in human and zoonotic or emerging pathogens. The possibility to play a major role in technology innovation and research consolidating our capacity in diagnostic, surveillance and epidemic preparedness is reinforced.

The launch of the NRCs promotes the public health microbiology domain as an essential tool to prevent and control infectious diseases in Belgium.

## Competing interests

The authors declare that they have no competing interests.

## Authors’ contributions

All authors contributed equally to this manuscript. GM responded to the comments of the referees and rewrote the article to its present form. All authors read and approved the final manuscript.
