# Correction: Establishment and reinforcement of the National Reference Centers for Human Microbiology in Belgium

**DOI:** 10.1186/0778-7367-71-8

**Published:** 2013-04-22

**Authors:** Gaëtan Muyldermans, Amber Litzroth, Geneviève Ducoffre, Sophie Quoilin

**Affiliations:** 1 Institute of Public Health, Public Health and Surveillance, J. Wytsmanstreet 14 1050, Brussels, Belgium

## Correction

The correct names of the authors of this article [1] are Gaëtan Muyldermans, Amber Litzroth, Geneviève Ducoffre, Sophie Quoilin and the Medical-Technical Advisory Board.

The Publisher and authors apologize to the readers for the inconvenience caused.

## References

[B1] Muyldermans G Litzroth A Ducoffre G Quoilin S the Medical-Technical Advisory Board Establishment and reinforcement of the National Reference Centers for Human Microbiology in Belgium Archives of Public Health 2012 70 16 22 June 2012 10.1186/0778-7367-70-16 22958353 PMC3436681

